# The soundscape of swarming: Proof of concept for a noninvasive acoustic species identification of swarming *Myotis* bats

**DOI:** 10.1002/ece3.9439

**Published:** 2022-11-14

**Authors:** Anja Bergmann, Lara S. Burchardt, Bernadette Wimmer, Karl Kugelschafter, Florian Gloza‐Rausch, Mirjam Knörnschild

**Affiliations:** ^1^ Museum für Naturkunde Leibniz Institute for Evolution and Biodiversity Science Berlin Germany; ^2^ Animal Behavior Lab, Freie Universität Berlin Berlin Germany; ^3^ Naturschutz, Landwirtschaft, Gartenbau, Schifffahrt und Wasserwirtschaft Landratsamt Garmisch‐Patenkirchen Garmisch‐Patenkirchen Germany; ^4^ ChiroTEC ‐ Verhaltenssensorik und Umweltgutachten Lohra Germany; ^5^ Noctalis Fledermaus‐Zentrum GmbH Bad Segeberg Germany; ^6^ Deutsche Fledermauswarte e.V Berlin Germany

**Keywords:** bats, echolocation calls, linear frequency cepstral coefficients, *Myotis*, noninvasive acoustic monitoring, noninvasive species identification, swarming phenology

## Abstract

Bats emit echolocation calls to orientate in their predominantly dark environment. Recording of species‐specific calls can facilitate species identification, especially when mist netting is not feasible. However, some taxa, such as *Myotis* bats can be hard to distinguish acoustically. In crowded situations where calls of many individuals overlap, the subtle differences between species are additionally attenuated. Here, we sought to noninvasively study the phenology of *Myotis* bats during autumn swarming at a prominent hibernaculum. To do so, we recorded sequences of overlapping echolocation calls (*N* = 564) during nights of high swarming activity and extracted spectral parameters (peak frequency, start frequency, spectral centroid) and linear frequency cepstral coefficients (LFCCs), which additionally encompass the timbre (vocal “color”) of calls. We used this parameter combination in a stepwise discriminant function analysis (DFA) to classify the call sequences to species level. A set of previously identified call sequences of single flying *Myotis daubentonii* and *Myotis nattereri*, the most common species at our study site, functioned as a training set for the DFA. 90.2% of the call sequences could be assigned to either *M. daubentonii* or *M. nattereri*, indicating the predominantly swarming species at the time of recording. We verified our results by correctly classifying the second set of previously identified call sequences with an accuracy of 100%. In addition, our acoustic species classification corresponds well to the existing knowledge on swarming phenology at the hibernaculum. Moreover, we successfully classified call sequences from a different hibernaculum to species level and verified our classification results by capturing swarming bats while we recorded them. Our findings provide a proof of concept for a new noninvasive acoustic monitoring technique that analyses “swarming soundscapes” by combining classical acoustic parameters and LFCCs, instead of analyzing single calls. Our approach for species identification is especially beneficial in situations with multiple calling individuals, such as autumn swarming.

## Graphical abstract

We provide the basis for a new noninvasive acoustic monitoring technique that analyses the echolocation calls of *Myotis* bats. Thereby, a combination of classical acoustic parameters and linear frequency cepstral coefficients makes the analysis of single calls obsolete. Our approach for species identification is especially beneficial in situations with multiple calling individuals, such as autumn swarming.

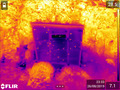

1

Autmn swarming in front of one of the two entrances of the Kalkberg cave at the 26th August 2019, 23:32. The video was recorded using a thermal camera (FLIR E95, Teledyne FLIR LLC, Wilsonville, USA).

## 2 INTRODUCTION

In our need to understand the behavior of animals, we often inadvertently affect it. Nevertheless, extensive monitoring is not only important for behavioral studies but also for conservation efforts. Without question, capturing animals facilitates information collection in terms of species identity, sex or age. In addition, bio‐loggers or tracking devices can be applied and provide information about an animal's internal state (reviewed in Cooke et al., 2004; Wilmers et al., 2015) or its external environment (Charrassin et al., 2002; Roquet et al., 2014). However, despite these advantages, capturing wild animals causes stress, which is especially relevant in the context of behavioral studies and the observation of rare and endangered species (Cattet et al., 2008; Lane & McDonald, 2010). To avoid interfering with the animals directly, noninvasive monitoring is a powerful tool to gain information about the natural behavior of wild animals or population dynamics. When the focal species are nocturnal, fast‐moving, or of small body size, visual observation becomes difficult and often is connected with high effort (Theriault et al., 2014). Therefore, depending on the focal species, its surroundings, and the goals of the observation, other techniques are applied, such as camera traps (Gilbert et al., 2021; Kalle et al., 2011), collection of feces (Kohn et al., 1999; Prugh et al., 2005) or acoustic monitoring (Enari et al., 2017; Oppel et al., 2014).

Acoustic monitoring is often used to detect bats, a crucial endeavor for conservation applications because more than half of all bat species occurring in Germany are endangered there and all are protected by national law (Meinig et al., 2020). In recent decades technical capabilities for detecting and analyzing bat sounds have developed rapidly (Grinnell et al., 2016), thus facilitating species identification via species‐specific echolocation calls emitted in‐flight. Those calls evolved for information acquisition, orientation and foraging in a predominantly dark environment (Griffin et al., 1958) and make bats capable of extraordinary spatial discrimination (e.g. Simmons et al., 1983). Besides encoding colony membership (Jameson & Hare, 2009; Masters et al., 1995), individual identity (Kazial et al., 2008; Voigt‐Heucke et al., 2010; Yovel et al., 2009), sex (Jones et al., 1992; Knörnschild et al., 2012; Siemers et al., 2005) or age (Jones et al., 1992; Masters et al., 1995), echolocation calls facilitate species recognition, even interspecifically (Schuchmann & Siemers, 2010). Indeed, differences in echolocation calls may reflect specific prey preferences or divergent foraging techniques, and often ecologically similar bats employ similar echolocation calls (Neuweiler, 2003; Schnitzler & Kalko, 2001; Siemers & Schnitzler, 2004). For instance, large similarities occur in the call structure of some European *Myotis* species, which are mainly adapted to orientation close to background vegetation. Such similarities complicate species identification through echolocation calls in this genus. Nevertheless, *Myotis* species are capable of discrimination between seemingly similar calls and even can recognize individual identity based on those (Kazial et al., 2008; Yovel et al., 2009), indicating the possibility of comprehensive species discrimination.

About 30,000 individuals of six different *Myotis* species hibernate at the Kalkberg cave in Northern Germany and among them are large numbers of Daubenton's bats, *Myotis daubentonii*, and Natterer's bats, *Myotis nattereri*, (estimations based on light barrier counts and camera traps; MELUND, 2019). Prior to hibernation *Myotis* bats and other temperate zone bats that hibernate in underground sites are often engaged in an activity known as “swarming.” Following the first observation in North America (Davis, 1964) also European bats were found to swarm at underground roosts outside the period of hibernation (Roer & Egsbaek, 1966). Swarming is characterized by intense flight activity, chase flights and circling in and around the entrances of the hibernacula (winter roosts used for hibernation) without entering, accompanied by a large amount of both echolocation calls and social vocalizations (Fenton, 1969; Parsons, Jones, & Greenaway, 2003). Behavioral and genetic studies have revealed various functions of swarming so far. Swarming is important to assess hibernacula, both for experienced individuals and their current offspring (e.g. Fenton, 1969; Stumpf et al., 2017). In addition, gene flow between otherwise isolated colonies and promiscuous mating behavior is facilitated when bats of different colonies meet at the swarming sites (e.g. Burns & Broders, 2015; Kerth et al., 2003; Rivers et al., 2005). Overall, swarming at hibernacula facilitates various, not mutually exclusive social functions depending on the individual's species, sex or age.

**FIGURE A1:**
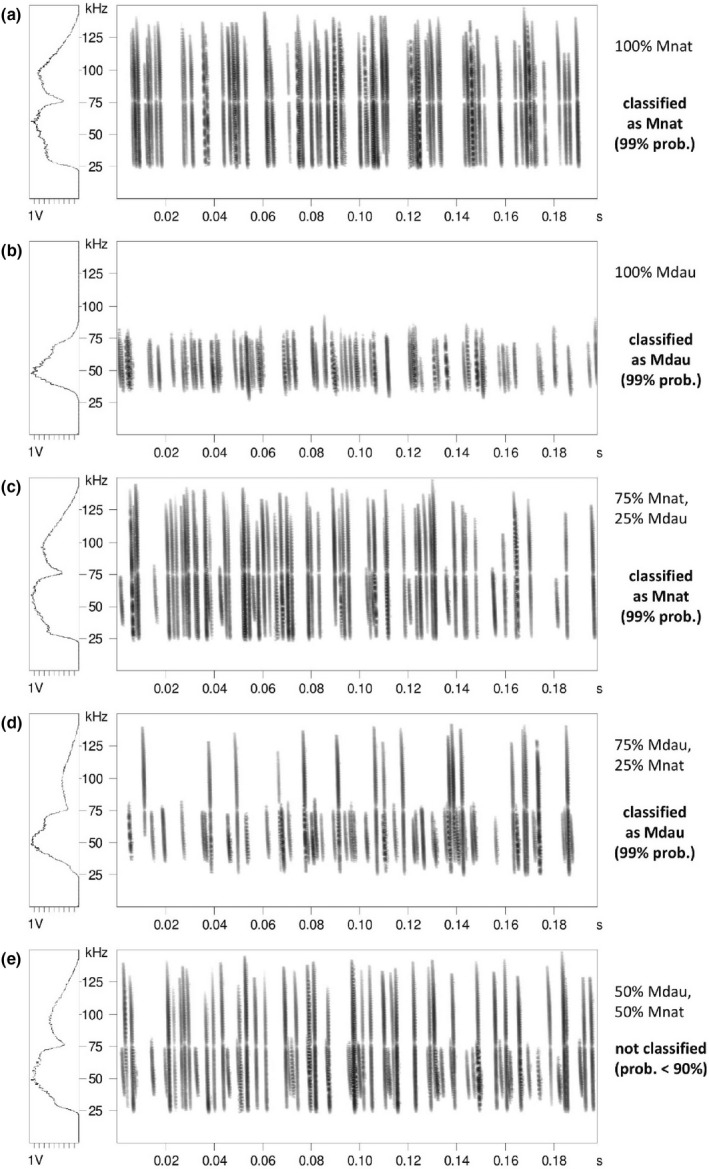
Visualization of the “swarming soundscape” analysis. Artificial “swarming” recordings were created by mixing 20 passes with known species ID (Mnat = *M. nattereri*, Mdau = *M. daubentonii*) in five different frequency ratios (a: 100% Mnat; b: 100% Mdau; c: 75% Mnat and 25% Mdau; d: 75% Mdau and 25% Mnat; e: 50% Mdau and 50% Mnat) and subsequently classifying their dominant species based on our reference data. Recordings could be classified correctly with the exception of the balanced mix (e). The classification probability of the balanced mix did not exceed our threshold of >90%, indicating that—as expected—the dominant species in this soundscape cannot be correctly identified.

Because swarming bats constantly emit echolocation calls, the calls strongly overlap, thus making small differences even more subtle and identification of some bat species very challenging (Rydell et al., 2017). While the acoustic species identification based on echolocation calls has made remarkable progress in recent years (Bas et al., 2017; Obrist & Boesch, 2018; Schwab et al., 2022), classifying the echolocation calls of many bats vocalizing at the same time (i.e. during swarming) still remains extremely difficult because it is often impossible to extract overlap‐free single calls or call sequences for species identification. Here, we demonstrate a proof of concept how this problem could be solved by focusing on the swarming soundscape, i.e. the predominant acoustic impression at a given time period during swarming instead of calls from single individuals (see Figure A1).

We discriminated two different *Myotis* species (*M. daubentonii* and *M. nattereri*) with the help of both classical acoustic parameters and derived parameters originally employed in human speaker recognition. Not only the spectro‐temporal structure of single calls but also the general sound characteristics of calls (such as color of voice) differ between species or even individuals, a fact that is for instance exploited in the speaker recognition algorithms of modern smartphones. Often, acoustic feature extraction techniques based on mel frequency cepstral coefficients (MFCCs) are used (reviewed in Jain & Sharma, 2013). MFCCs use a mel scale, which is linear up to 1 kHz and logarithmic above to emphasize low frequencies, like human voice. For signals with a higher frequency, such as echolocation calls, this emphasis is not desirable and a linear scale can be applied yielding linear frequency cepstral coefficients (LFCCs) instead (Zhou et al., 2011). Both cepstral coefficients make the measurement of single call parameters expendable by representing entire signals in a compact form. During the process of feature extraction, the information of the whole signal is condensed in several steps of calculations (Cuong et al., 2012; Loughran et al., 2008). Cepstral coefficients in combination with classical acoustic parameters (e.g. peak frequency, duration, etc.) have been employed to facilitate species identification based on single calls for crickets and katydids (Noda et al., 2019) or fish (Noda et al., 2016). Furthermore, cepstral coefficients have been used to categorize call types of giant otters groups (Mumm & Knörnschild, 2017) or to discriminate between colony‐specific signatures in territorial songs of male bats (Knörnschild et al., 2017).

The goal of our study was to test whether our approach would allow us to identify two swarming *Myotis* species based on the soundscape their echolocation calls created. We presumed that we could identify the predominantly swarming species during a given time period by comparing our recordings of overlapping echolocation call sequences (swarming soundscapes) to a set of reference data, i.e., previously identified echolocation call sequences, thus making the analysis of single call sequences obsolete. We used the second set of previously identified call sequences to validate our classification results. Moreover, we compared our acoustic species identification of swarming bats to the known swarming phenology of both species at our study site.

## 3 METHODS

### 3.1 Study site and bat activity

Recordings of echolocation calls were conducted at the Kalkberg cave (10°18′57′′E; 53°56′09′′N), one of the most important hibernacula of bats in central Europe. The natural cave is located in Bad Segeberg, Northern Germany, and shelters more than 30,000 bats per winter (MELUND, 2019). Both entrances of the cave have been monitored with light barriers (ChiroTEC, Lohra, Germany) since 1991, counting incoming and departing individuals. Among the hibernating bats are six *Myotis* species, with *M. nattereri* and *M. daubentonii* making up for about 90% of the winter population (estimations based on light barrier counts and camera traps; MELUND, 2019). In total, at least seven bat species use the Kalkberg cave: *M. nattereri*, *M. daubentonii*, *M. brandtii/mystacinus*, *M. bechsteinii*, *M. dasycneme*, *M. myotis*, and *Plecotus auritus* (sorted from common to rare; MELUND, 2019). Prior to hibernation, between August and November, the vicinity of the cave is extensively used for autumn swarming (Video 1). In addition, we recorded swarming bats at another site in Northern Germany (Lüneburg) while simultaneously capturing bats with mist nets in the direct vicinity of their swarming site.

### 3.2 Acoustic recordings and data preparation

We employed a total of three data sets consisting of echolocation call sequences for the analyses. The first data set (test data A and B) contained recordings of overlapping echolocation call sequences of swarming bats in front of the Kalkberg cave (A) and the second site in Northern Germany (B).

We wanted to identify the predominantly echolocating species in these recordings with the help of a second data set (reference data), which contained echolocation call sequences of *M. daubentonii* or *M. nattereri* in a single flight, assigned to species level via photos from synchronized camera trap images. A third data set (control data) contained additional previously identified echolocation call sequences of both focal species from single flights. We used the reference data as training data in a discriminant function analysis (DFA) to classify recordings from the test data and the control data as either *M. daubentonii* or *M. nattereri*. Due to the difference in recording quality, recordings from the three data sets were in part prepared differently for subsequent analyses (for details, see below).

#### 3.2.1 Test data: unidentified echolocation call sequences of swarming *Myotis* bats

**FIGURE 1:**
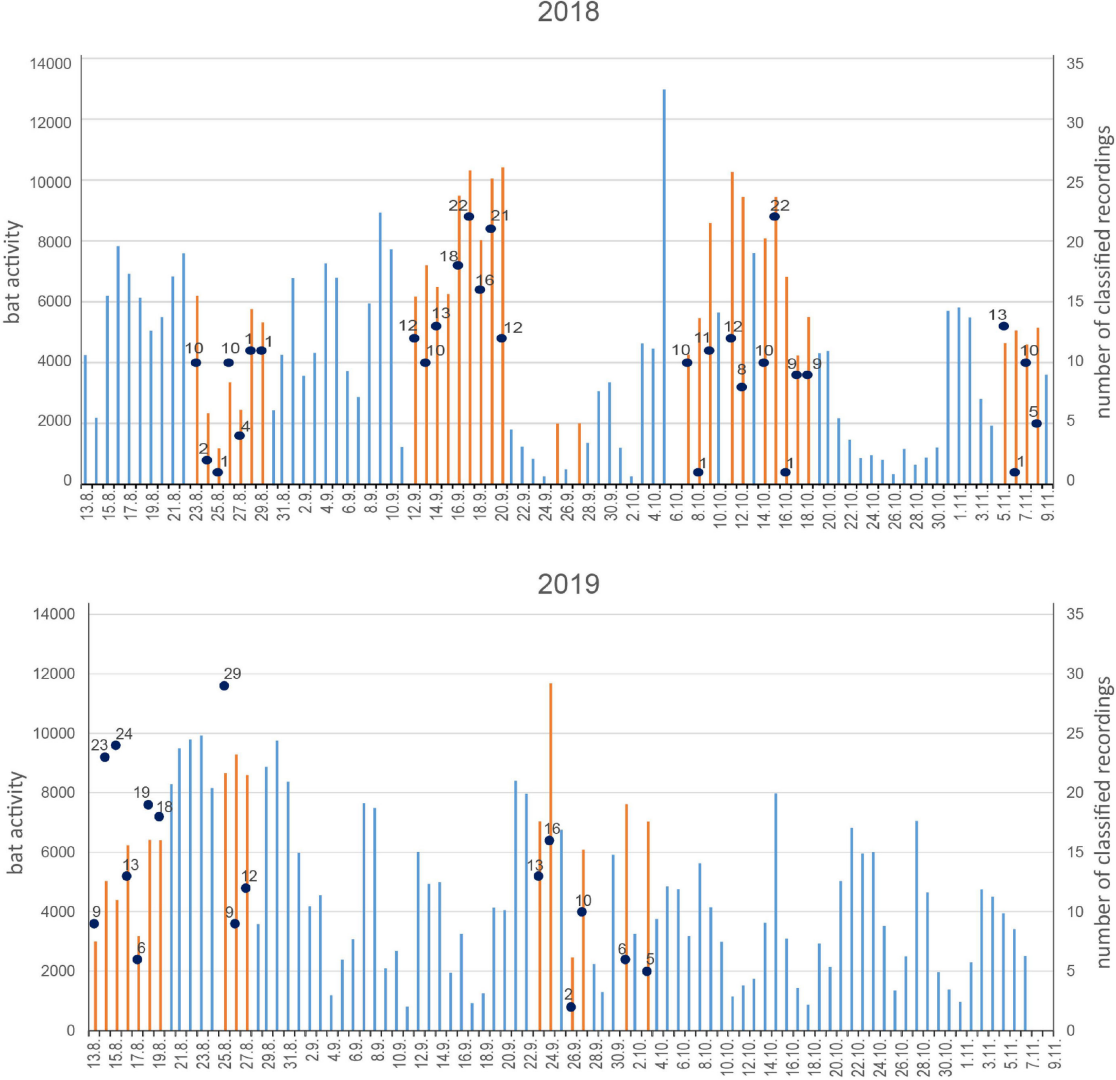
Total activity (sum of arrivals and departures counted via light beam interruptions) of bats at the hibernaculum per night during the swarming seasons of 2018 and 2019. The nights during which sound recordings were conducted are highlighted in orange. Recordings started in mid‐August and continued until mid‐November. Numbers indicate the amount of analyzed echolocation call sequences per recording night, which were classified as *Myotis daubentonii* or *Myotis nattereri* with a classification probability of 90% or higher.

Recordings were conducted during 45 nights in two consecutive swarming seasons (August to November 2018 and August to October 2019) at various times between sunset and sunrise, mainly during the highest swarming activity (2 h after sunset until 2 am) at both entrances of the Kalkberg cave (test data set A). Recording sessions were initiated based on the local weather at the beginning of the night (no rainfall, mild temperatures, little wind, i.e., Beaufort Force 0–3). As weather conditions sometimes changed drastically during the night, the recording nights were not always the nights with the highest swarming activity. During the recording nights in 2018, the maximum activity (sum of arrivals and departures counted via light beam interruptions) was 10,415 and the minimum activity was 1182 (Figure 1). During the recording nights of 2019, the maximum activity was 11,678 and the minimum activity was 2162.

Acoustic recordings were made whenever a high number of individuals was swarming simultaneously (observed with a thermal video camera; FLIR E95, Teledyne FLIR LLC, Wilsonville, USA). We are aware that this selection of specific recording situations may cause a bias in our data set (e.g., rarer species may only swarm when it is less crowded) but our first priority was to test whether our approach works during high swarming activity with many overlapping calls (proof of concept). Echolocation call sequences were recorded (sampling rate 500 kHz, 16‐bit depth resolution) using a high‐quality ultrasonic microphone (Avisoft USG 116Hm with condenser microphone CM16; frequency range 1–200 kHz) connected to a small computer (Dell Venue 8) running the software Avisoft Recorder (v4.2.05, R. Specht, Avisoft Bioacoustics, Glienicke, Germany). For the subsequent acoustic analysis, 564 echolocation call sequences (mean: 11.3 sequences per night; range: 1–29) with a length of 4 s each were selected based on the quality of the sound recordings and the presence of a high number of echolocation calls without interfering social vocalizations. Again, this choice may have caused a bias in our data set (e.g. some species may produce many social calls during swarming and would thus be less represented in our data set) but it was unavoidable because we did not have a training set of social calls identified to species level to complement our training set of echolocation calls. The selected call sequences were band‐pass filtered (15–150 kHz) and amplified digitally by 6 dB in Avisoft‐SASLab Pro (v5.2.13, R. Specht, Glienicke, Germany) prior to further analysis.

We also recorded swarming bats during one night (22.09.2021) at another site in Northern Germany (Lüneburg) and simultaneously captured swarming bats with mist nets located 2 meters away from the microphone (test data set B). Recordings were made, selected, and subsequently processed as described above. Data set B was much smaller than data set A, comprising only 30 echolocation call sequences with a length of 4 s each but valuable because the dominant species of the swarming bats was confirmed by simultaneous capture (87% of captured bats were *M. nattereri*).

#### 3.2.2 Reference data

**FIGURE 2:**
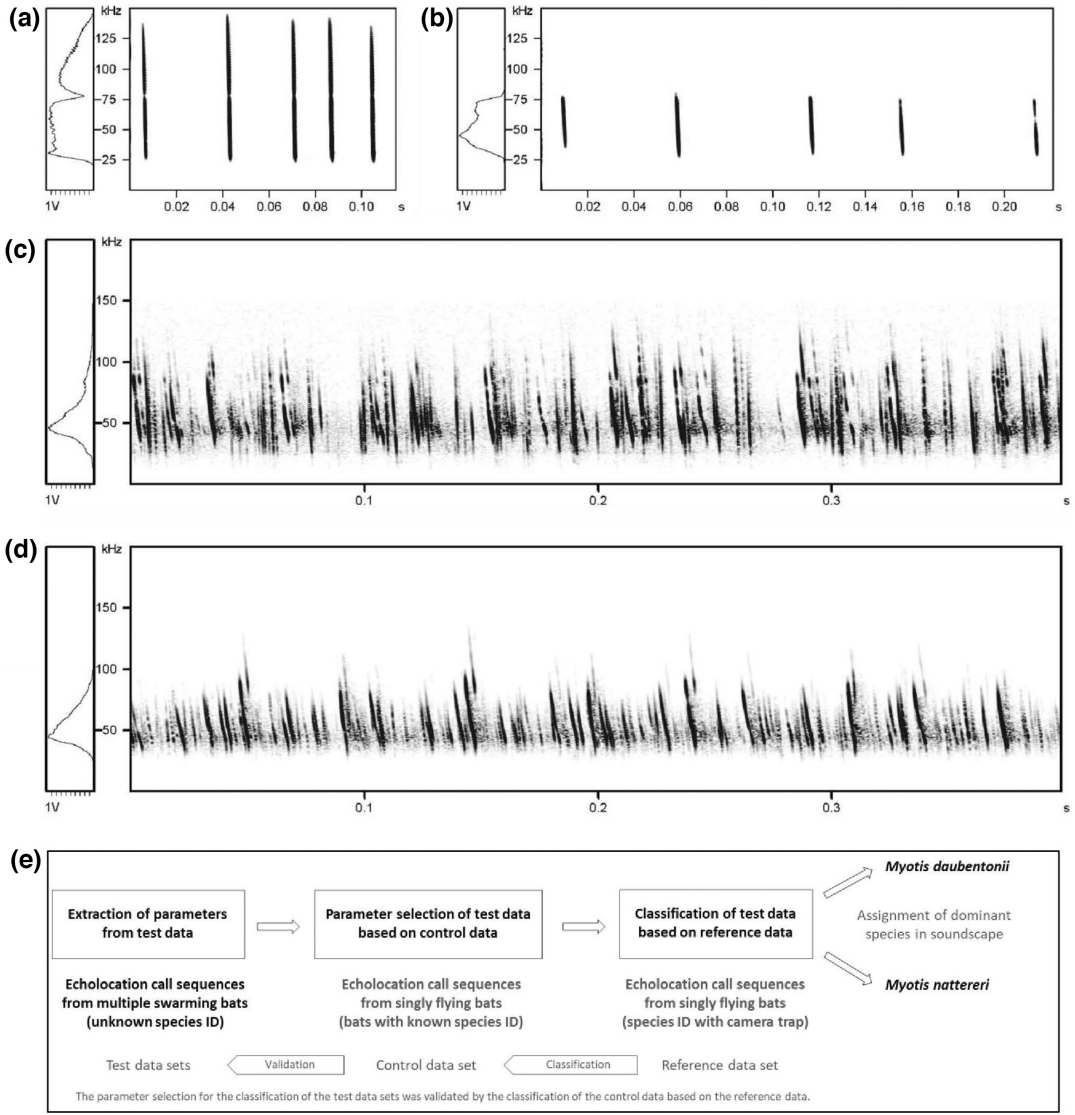
Reference calls of *Myotis nattereri* (a) and *Myotis daubentonii* (b) were used to classify recordings with unknown echolocation calls (c and d). The employed sequences consisted of five consecutive echolocation calls without background noise. Based on the reference data set, recordings with overlapping echolocation calls from a swarming situation were classified as predominantly *M. nattereri* (c) or *M. daubentonii* (d). The bottom panel (e) illustrates our classification procedure and how reference, control and test data sets are connected. Spectrograms were created using a 1024 FFT and a Hamming window with 87.5% overlap. See Figure A1 for a visualization of the “swarming soundscape” analysis.

To classify the recorded echolocation call sequences from the swarming situation, identified echolocation call sequences of *M. daubentonii* and *M. nattereri* were used as a reference (i.e. as training set in a discriminant function analysis). These echolocation call sequences came from singly flying individuals and were recorded at 10 underground sites with a Batcorder (ecoObs GmbH, Nürnberg, Germany) using a sampling rate of 500 kHz and a trigger threshold of −36 dB (quality 26–28). The calling species were identified via photos from synchronized camera trap images (Wimmer & Kugelschafter, 2015): Whenever bats were flying through a narrow underground passage, a light barrier was interrupted, which triggered a sound recording and a corresponding photo from a camera trap (ChiroTEC, Lohra, Germany). If it was possible to identify the species of the calling bat based on the photo, the respective recording was saved in a database. From these recordings, we selected 60 sequences of five high‐quality echolocation calls per species, *M. daubentonii* and *M. nattereri*, for further analysis (Figure 2). Selected sequences were 0.1–0.3 seconds long. Prior to acoustic analyses, the noise was reduced by 50 dB, high‐pass filtering was applied (25 kHz) and the volume of the calls was raised by 6 dB in Avisoft‐SASLab Pro. To avoid treating the background noise like a signal during the feature extraction (details below), it had to be eliminated prior to further analysis. For this purpose, we deleted all temporal gaps between echolocation calls in the reference data. Even though in the reference data echolocation calls of *M. daubentonii* were often multi‐harmonic in structure (due to the very small distance of bats to the microphone at the underground sites), only the first harmonic (fundamental frequency) was used for acoustic analyses. The second harmonic is not recorded when *M. daubentonii* is echolocating at a distance (Britton & Jones, 1999; Schaub & Schnitzler, 2007), as it was the case for our recordings from the Kalkberg cave (test data set A) and Northern Germany (test data set B).

#### 3.2.3 Control data

To validate our statistical classification of the test data sets A and B, we classified an additional data set as a control using the same reference data. For this control data set, the species identity of the calling bats was also deduced unequivocally, e.g., when bats were recorded flying near their roost and the species composition of the roost was fully known. The echolocation call sequences in the control data set were recorded using a Petterson D980 (Pettersson Elektronik AB, Sweden) in time expansion mode (Skiba, 2009). All sound recordings were stored on magnetic tape and digitized at the Museum for Natural History, Berlin (300 kHz, 16 bit). We selected 16 high‐quality echolocation call sequences of *M. daubentonii* and *M. nattereri*, respectively. These sequences consisted of 4–7 consecutive echolocation calls each and were 0.4 s long; they were slightly longer than the sequences in the reference data set because of larger inter‐call intervals. The control data were used to validate our statistical classification (i.e., the selection of acoustic parameters to discriminate between *M. daubentonii* and *M. nattereri*) and treated in the same way as the reference data (noise reduction, high‐pass filtering, volume change, gap removal) prior to acoustic analyses. It was not necessary to remove the second harmonic for echolocation calls of *M. daubentonii* in the control data set because only the first harmonic was recorded (as it is normally the case for field recordings).

### 3.3 Acoustic analysis

In total, we extracted 14 acoustic parameters for a general description of calls and subsequent statistical analysis, four spectral parameters (start, end and peak frequency, spectral centroid) and 10 derived acoustic parameters (mean and standard deviation for LFCC 1 to 5).

Spectral parameters: Start, end, and peak frequency for the test data sets A and B were calculated with a custom‐made MATLAB routine over the entire file in 10 ms frames using the *meanfreq* function from the Signal Processing toolbox. The analysis of single calls was not possible in the test data sets A and B (swarming bats) because there was much overlap between calls in the sequences. For the reference and the control data sets, start, end, and peak frequency of all echolocation calls in a sequence were measured in Avisoft‐SASLab Pro (threshold of −24 dB relative to the peak amplitude; values averaged over the entire call). In contrast to the test data, single calls were measured in the reference and control data sets. For all data sets, we also calculated the spectral centroid of each echolocation call sequence in Avisoft‐SASLab Pro (threshold: −28 dB relative to peak amplitude).

Linear‐frequency cepstral coefficients (LFCCs): We additionally used an acoustic feature extraction technique based on LFCCs for all data sets. As spectral‐based representations of entire signals, LFCCs capture the most important features of a signal in a compact form. For all data sets the feature analysis was run with a custom‐made routine in the speech processing toolbox “voicebox” in MATLAB (v. R2018b). In total, five LFCCs were extracted (Hamming window; test data: 100 ms frame; reference and control data: 3 ms frame). Subsequently, values for each frame were summarized by calculating the mean and standard deviation for each of the five features for every analyzed echolocation call sequence.

### 3.4 Statistical analysis

To test for species identity (i.e., the identity of the dominant species in each recording; see Figure A1), we performed stepwise discriminant function analyses (DFA) with subset validation, in which the reference data (with known species ID, 120 sequences) functioned as the training set. In the first DFA, the control data (also with known species ID, 32 sequences) was used to validate our statistical approach and select the acoustic parameters most important for the correct species identification. Resulting from this, the second DFA was applied to classify the test data set A (564 sequences with unknown species ID) using the parameters spectral centroid, start frequency, peak frequency, and mean and standard deviation of the LFCCs 1 and 3. We selected those parameters because they were the most important ones for correctly classifying the control data, as indicated by an initial stepwise DFA (end frequency, LFCC 2, 4, and 5 were excluded by the analysis). A third DFA was conducted with the same selection of acoustic parameters to classify the test data set B associated with simultaneous bat capture (30 sequences with unknown species ID). Prior to the analyses, we checked our data for multivariate normality and homogeneity of variances/covariances. Statistical tests were conducted using SPSS (version 20, SPSS Inc., Chicago, IL, USA).

## 4 RESULTS

### 4.1 Control data were classified correctly

Using seven acoustic parameters (start and peak frequency, spectral centroid, mean and standard deviation of LFCC 1 and 3) in a stepwise DFA with subset validation, the species ID of all 32 echolocation call sequences in the control data set could be classified correctly with a minimum classification probability of 94% (DFA: Training *N* = 120, Test *N* = 32, Eigenvalue = 12.225, explained variation = 100%, *Wilk’s λ* = 0.076, *χ* ^*2*^ = 295.648, *p* < .0001). The same parameters were afterward employed in the second and third DFA with unidentified echolocation call sequences from swarming bats as test data sets.

### 4.2 Most call sequences from swarming bats could be classified to species level

The test data set A contained 564 sequences (4 s each) of overlapping echolocation calls of multiple swarming bats. With the selected seven parameters described above, we could classify the vast majority of the recordings (509 sequences, 90.2%). Out of the 564 call sequences, 184 were classified as *Myotis daubentonii* and 325 as *Myotis nattereri* with a classification probability of 90% or higher (DFA: Training *N* = 120, Test *N* = 564, Eigenvalue = 12.225, explained variation = 100%, Wilk’s *λ* = 0.076, *χ* ^2^ = 295.648, *p* < .0001, see also Table A1 and A2). The other 55 echolocation call sequences had a lower classification probability and were thus discarded from further analysis.

### 4.3 Differences in the parameter distribution of all data sets

**FIGURE 3:**
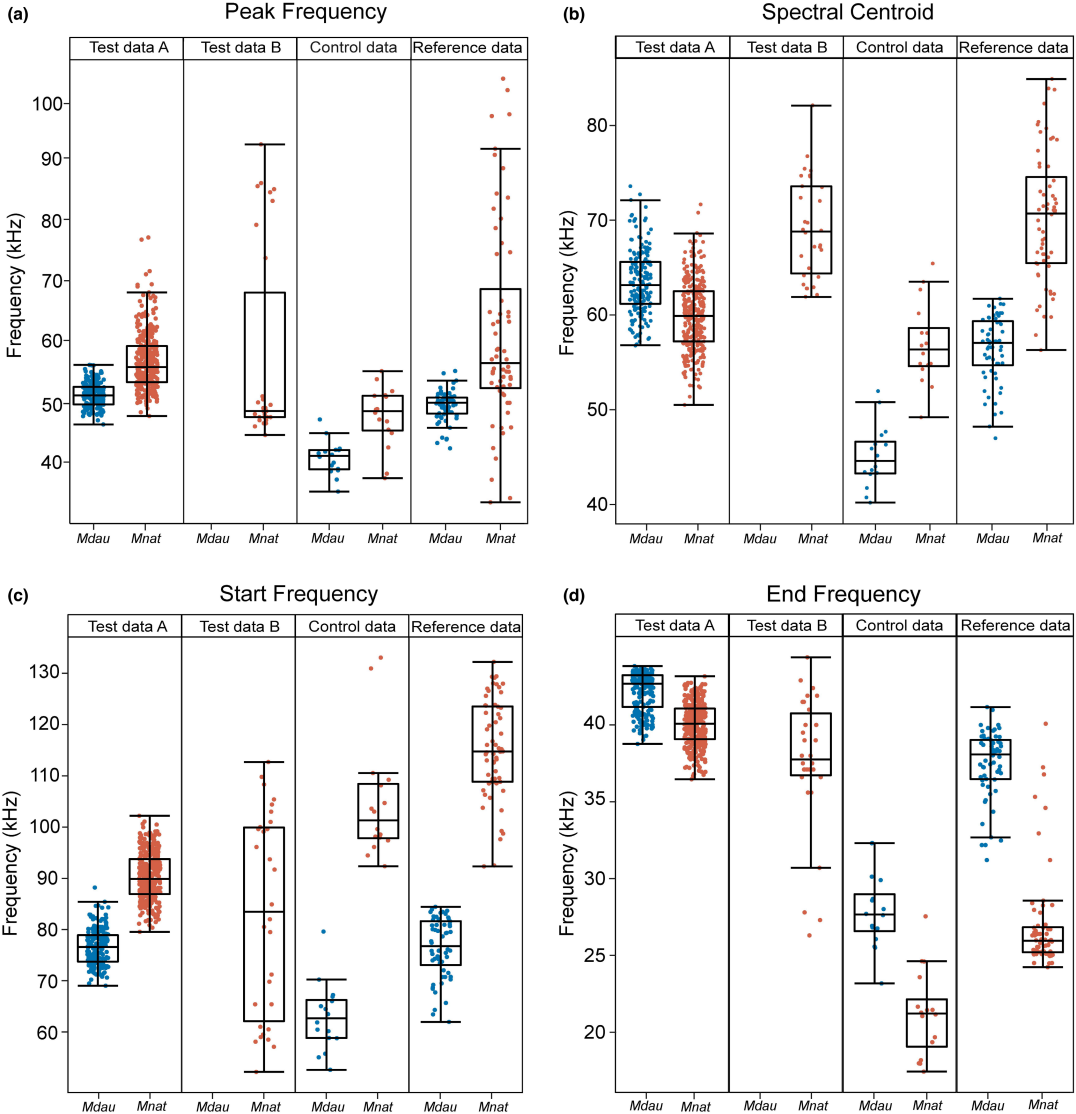
To classify unknown echolocation call sequences, the classical acoustic parameters peak frequency (a), spectral centroid (b) and start frequency (c) were used in addition to linear frequency cepstral coefficients (see Figure A2). The end frequency (d) was excluded from further analysis because this parameter was not crucial for discriminating the control data based on the reference data. For the control and reference data the species identity was known before. The species identification of the test data was based on our analysis. All recordings from test data set B were classified as *M. nattereri*. Mdau = *Myotis daubentonii*; Mnat = *Myotis nattereri*.

**FIGURE A2:**
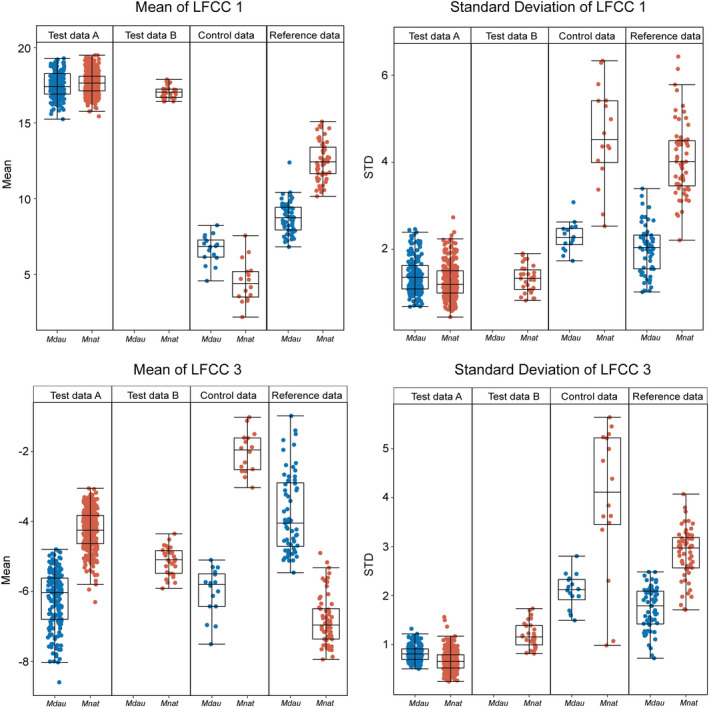
Distribution of mean and standard deviation of LFCC 1 and 3 for all data sets. For the control and reference data, the species identity was known before. The species identification of the test data sets A and B was based on our analysis (and, for set B, validated by simultaneous bat capture). Mdau = *Myotis daubentonii,* Mnat = *Myotis nattereri*.

The distribution of the extracted classical acoustic parameters differed between all data sets (Figure 3). The largest differences between species were visible in the start frequency. For all data sets containing both species, the start frequency of *M. nattereri* was higher than that of *M. daubentonii*. However, in the test data set A, the differences were more subtle and both species' start frequencies overlapped between 80 and 85 kHz. The peak frequency of *M. nattereri* varied considerably in the reference data and test data set B, while the ranges were lower in the other data sets and also for *M. daubentonii*. Nevertheless, in all data sets the median of the peak frequency of *M. nattereri* was higher than that of *M. daubentonii*. For peak and start frequency all data sets displayed the same relation. Also, in the excluded end frequency the relation between species was the same in all data sets, even though the difference between species in the test data set A was the smallest. By contrast, the spectral centroid was the only parameter for which the distribution of the test data set A, and both the reference and the control data were opposed: for *M. daubentonii* it was higher in the test data set A but lower in the reference and control data. The inconsistent pattern for the spectral centroids may have been caused by the fact that the test data set constituted a much more chaotic acoustic situation than the other data sets (many bats from two different species echolocating simultaneously). The distribution of the extracted acoustic features (LFCCs) can be found in the Figure A2; those values were much less scattered than the original acoustic parameters.

For all three classic acoustic parameters (start, peak, and end frequency), the frequency values of the two focal species differed less during swarming (test data) than during single flight close to clutter (control and reference data). As the test data sets A and B were recorded in very crowded swarming situations, the probability is high that in each sequence of 4 s, more than one species was present. Thus, both species' echolocation calls influence the frequency distributions while our classification results emphasize only the predominant species.

### 4.4 Classification results reflect known swarming phenology

**FIGURE 4:**
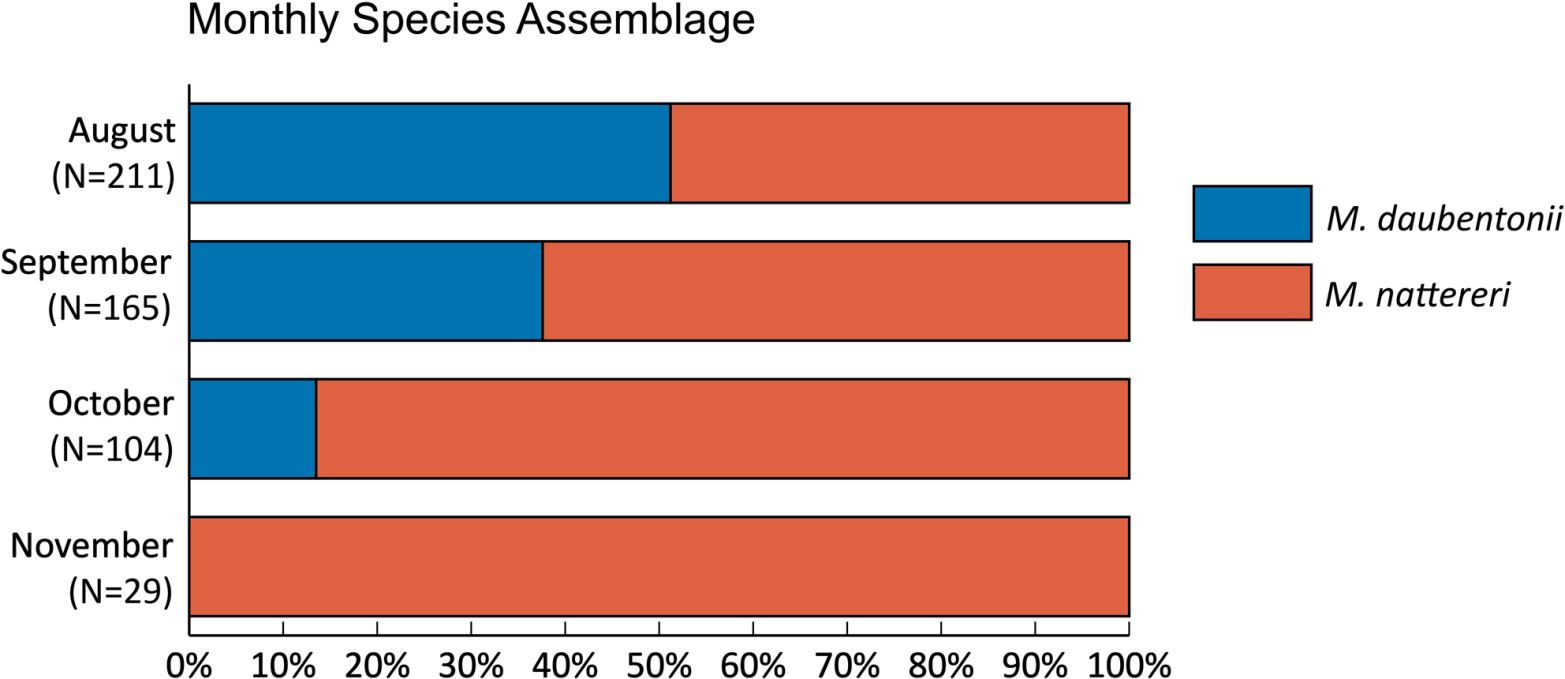
Monthly species assemblage in the course of the swarming season, based on the analysis of the echolocation calls of swarming bats from 2018 and 2019. The known phenology of both species is reflected in the species assemblage. With a classification probability of 90% or higher 184 sequences were classified as *Myotis daubentonii* and 325 as *Myotis nattereri*, 55 call sequences were discarded due to a lower classification probability.

Previous studies and intense monitoring and mist netting over several years indicate that at the Kalkberg cave *M. daubentonii* swarm from August onwards and immigrate into the hibernaculum from mid‐September to the end of October. In September multiple nights are clearly dominated by swarming *M. nattereri*, which immigrate into the hibernaculum from mid‐October to the end of November (Kugelschafter, 1999, 2000, 2001). This well‐documented swarming phenology is also reflected in our classification results, thus further validating them. In August, the echolocation call sequences were equally classified as *M. daubentonii* and *M. nattereri*. In the subsequent months, the proportion shifted in favor of *M. nattereri* (62% in September, 85% in October) until they made up for 100% in November (Figure 4).

### 4.5 Classification results correspond to the species ID of bats captured while recording

As an additional validation, we used the reference data to classify the test data set B (30 sequences, 4 s each), which was obtained while simultaneously capturing bats with mist nets in direct vicinity of the swarming site (mist nets were placed 2 m away from the microphone). This data set was not recorded at the Kalkberg but at a different swarming site in Northern Germany. In total, we captured 349 bats, 304 *M. nattereri* (87%) and 42 *M. daubentonii* (12%); the remaining bats (1%) were 2 *M. myotis* and 1 *M. bechsteinii*. Correspondingly, all recordings were classified as *M. nattereri* (DFA: Training *N* = 120, Test *N* = 30, Eigenvalue = 12.225, explained variation = 100%, Wilk’s *λ* = 0.076, *χ* ^2^ = 295.648, *p* < .0001, see also Table A1 and A2). For details on the acoustic parameters of the recordings, please see Figure 3 and Figure A2.

## 5 DISCUSSION

Identifying swarming bats noninvasively is challenging, but we were able to assign echolocation call sequences of swarming *Myotis* bats to species level based on a combination of classical acoustic parameters and linear frequency cepstral coefficients (LFCCs), thereby indicating the predominant species. The combined use made analyzing single calls obsolete — which often is impossible during swarming, anyway — and enabled us to distinguish between two *Myotis* species in a swarming context. Some *Myotis* species in Germany employ rather similar echolocation calls, thus making them difficult to distinguish acoustically (Rydell et al., 2017), even in otherwise ideal recording situations (Wimmer & Kugelschafter, 2015). However, we focused on two species with a more distinct call design than others in the genus *Myotis*, which probably explains our satisfactory classification results. Future studies are needed to investigate how well our approach would work for other, acoustically more similar *Myotis* species.

Our statistical classification of echolocation call sequences corresponds well to the known swarming phenology of *M. nattereri* and *M. daubentonii* at our study site (Kugelschafter, 1999, 2000, 2001; MELUND, 2019). In contrast to hibernacula located in the UK (Parsons, Jones, et al., 2003; Rivers et al., 2006), the Netherlands and Belgium (van Schaik et al., 2015), a high proportion of *M. nattereri* were already present in August and September. It is unclear whether this is a regional difference in swarming phenology or caused by the fact that the Kalkberg cave is one of the largest hibernacula for *M. nattereri* in Central Europe and is also used as a summer roost for males (MELUND, 2019). Our statistical classification of echolocation call sequences also corresponds to the species ID of swarming bats captured while recording at another site. This indicates that acoustic monitoring is a suitable alternative or valuable addition to more invasive methods for species identification during swarming.

Combining classical acoustic parameters and LFCCs can enhance the success of bat species identification in situations that have been challenging in the past and can help to make the most of acoustic monitoring. The combination of classical acoustic parameters and cepstral coefficients has led to convincing classification results for other species such as fish (Noda et al., 2016) and insects (Noda et al., 2019) and it has also been used to discriminate between individuals or contexts (giant otters: Mumm & Knörnschild, 2017; bats: Araya‐Salas et al., 2020; Fernandez & Knörnschild, 2020; Knörnschild et al., 2017). In contrast to our test data, all those studies are based on sound recordings containing vocalizations of one individual or one particular species. As we could prove by identifying the control data correctly, our approach also works for the analysis of single calls. However, the necessary amount of postprocessing is higher for the analysis of single calls, because the gaps between calls have to be removed to minimize the influence of background noise. In comparison, sequences of multiple calls of swarming bats in the test data made the influence of background noise neglectable for LFCC extraction. Thus, less postprocessing is required for recordings of swarming bats, making our method best applicable to recordings of multiple overlapping calls. As the amount of postprocessing and analyzing time hardly increases with a higher number of recordings, monitoring over several nights or the whole season is easily feasible, making our approach suitable for long‐term monitoring of large bat groups. For future studies, it would be best to use an automatic recording device that is permanently installed at the swarming site and randomly select a fixed number of recordings per night for subsequent analysis. This fine‐scaled approach may enhance our understanding of the species‐specific phenology at swarming sites.

The main benefit of our approach is minimizing disturbances of hibernating bats by applying noninvasive acoustic monitoring techniques prior to hibernation. Mist netting at mass hibernacula during swarming can impact the animals and lead to disturbances of the natural behavior. Also, in demanding environments such as cliffs mist netting of bats often is not an option for species identification. In such scenarios, noninvasive acoustic monitoring shows its major advantages, as the effort of acoustic recording is comparatively low and it can be conducted over several nights during the season near hibernacula without affecting the animals. However, our approach has caveats as well: it is currently not possible to gain information on the presence of swarming species that occur in low numbers (because focusing on soundscapes will only identify the most dominant species in a recording), and even if a species is abundant enough to be detected, it is difficult to determine the species‐specific onset and cessation of activity for the species that are not dominating the recordings. This severely limits our ability to understand swarming patterns (size, species assemblage, annual occurrence, etc.), which are crucially needed to improve species conservation in the long term. Our approach currently represents a proof of concept, showing that it is possible to classify recordings made during swarming based on the soundscape that the predominantly echolocating species creates.

Another application possibility is the identification of so far understudied social calls emitted by bats on the wing during autumn swarming. Species information about in‐flight social calls of European *Myotis* bats is scarce, especially in a swarming context (Pfalzer, 2002; Pfalzer & Kusch, 2003), and the same is true for North American bats (Bohn & Gillam, 2018). We assume that it should be possible to identify social calls to the species level based on the surrounding echolocation calls.

Overall, our introduced noninvasive approach simplifies species identification especially in demanding environments and in situations with many calling individuals such as swarming. So far, we are able to acoustically separate two swarming *Myotis* species based on a set of reference data containing identified call sequences of both species. With additional high‐quality reference data sets for other species, our approach should be easily adaptable to identify more than two species, which is especially important for hibernacula with a more diverse species assemblage. Ultimately, we aspire to the application of our approach at swarming sites with so far unknown attendees to gain information about new autumn swarming sites and thus hibernacula. The more we know about species assemblage, phenology, and overall behavior at swarming sites, the better we will be able to protect endangered bat species and their hibernacula in the future.

## AUTHOR CONTRIBUTIONS

**Anja Bergmann:** Conceptualization (equal); data curation (equal); formal analysis (equal); investigation (equal); methodology (equal); visualization (equal); writing – original draft (lead). **Lara Sophie Burchardt:** Formal analysis (equal); software (equal); writing – review and editing (equal). **Bernadette Wimmer:** Investigation (equal); writing – review and editing (equal). **Karl Kugelschafter:** Investigation (equal); writing – review and editing (equal). **Florian Gloza‐Rausch:** Conceptualization (equal); investigation (equal); supervision (equal); writing – review and editing (equal). **Mirjam Knoernschild:** Conceptualization (equal); formal analysis (equal); investigation (equal); methodology (equal); software (equal); supervision (equal); writing – review and editing (equal).

## CONFLICT OF INTEREST

The authors declare that the research was conducted in the absence of any commercial or financial relationships that could be construed as a potential conflict of interest.

## FUNDING INFORMATION AND PERMITS

This work was supported by stipends from the Elsa‐Neumann Foundation to Anja Bergmann and Lara Burchardt. The investigations were conducted under license LLUR_521_20180703 (Bad Segeberg) and 61.24–61.22.50 (Lüneburg).

## Data Availability

The sound recordings used in this study are available on Dryad https://doi.org/10.5061/dryad.wdbrv15s8.

## APPENDIX A

**TABLE A1:** Assessment of model fit of all discriminant function analyses

Function	Eigenvalue	% of variance	Canonical Correlation	Test of function	Wilkins *λ*	*χ*2	df	*p*
1	12.225	100	0,961	1	0,076	295,648	7	<.0001

**TABLE A2:** Structure matrix showing the canonical loading (indicating the contribution of different acoustic parameters to the discriminant function) of the discriminant function for the seven acoustic parameters included in the stepwise discriminant function analyses.

Acoustic parameter	df 1
peak frequency	−0.019
start frequency	1.515
spectral centroid	−0.739
LFCC_MEAN_1	0.524
LFCC_STD_1	0.238
LFCC_MEAN_3	0.423
LFCC_STD_3	0.126
